# Remarks on the Third Stage of Labor

**Published:** 1851-09

**Authors:** J. G. House


ART. IV.—*Remarks on the Third Stage of Labor.* By J. G. HOUSE, M. D.

In presenting some remarks on the third stage of labor, I do not expect to enlighten my seniors in the profession, but may, perhaps, prove of some little service to my younger brethren who are assuming so responsible a duty as the obstetrical practice.

My inducements have been mainly these, viz., *first,* the importance of the subject; and, *secondly,* because several articles on manual delivery of the placenta have appeared in the journals, whose teachings have seemed to me to be wrong, and even dangerous when carried into practice.

Most experienced accoucheurs will probably agree that the time when they feel the most solicitude for the welfare of their patient, in the great majority of cases, is during the third stage of labor. It is then that the woman is specially liable to sudden exhausting hæmorrhages, which may immediately extinguish life, or else be followed by a train of formidable symptoms — the result of anaemia, or a portion of the secundines may be left within the cavity of the uterus, causing putrid absorption and adynamic fever. Hence the proper management of this part of the delivery assumes a vast importance, and frequently calls for all the judgment, knowledge, and firmness which the intelligent practitioner possesses.

In the articles above alluded to, the writers have advocated, in all cases, when the secundines have not been expelled from the uterus in five or ten minutes, an introduction of the hand into the same, and an immediate delivery of its contents, and have averred that the practice was safe and proper.

In the following remarks I shall endeavor to prove that the course is often very improper, worse than useless, and in many cases hazardous.

Natural labor should always be considered a physiological process — a natural result, like the falling of the ripe fruit from the tree, or the bursting of the fully matured pod and the discharge of its seed. The attendant has only to stand by, and witness the operation. I fear that he who advocates interference forgets that he is attending a normal, not an abnormal action; besides, the very etymology of his name, obstetrician, means to stand by. It is his duty, then, to stand by, and see that nature is not thwarted in her operations, and, in such case to act promptly, and only then. Let us examine some of the physiological phenomena of labor. That hollow muscle, the uterus, together with the whole voluntary system of the mother, have just been making a tremendous, and almost superhuman effort in expelling the fœtus and "tired nature seeks repose." The uterus seems as if conscious of having performed a great act, and to require time to collect and equalize her forces for a succeeding undertaking. Now unless some accident calls for interference, she should be allowed this respite, be the same ten minutes or thirty. If granted this repose, the natural contractions will, in forty-nine cases out of fifty, expel the placenta from the womb, and it will be found in the vagina ready to be discharged. Blundell says "a meddlesome midwifery is always bad;" that is, all interference with a healthy labor in any of its stages is injurious, so I understand the aphorism. But why is it bad in the stage under consideration? I will try to answer the question.

All irritation of the *os tincæ* either by frequent examinations, or pulling at the funis while the muscular fibres are suffering from fatigue, tend to excite irregular and spasmodic contractions of that organ; hence we find that we thereby produce premature efforts at delivery. The uterus then assumes various shapes, sometimes being elongated, lobulated, or one lateral half of the organ contracted, while the other remains stationary, or the anterior half contracted, and posterior expanded, or, finally, the circular fibres acting entirely, or in part, and the longitudinal quiescent. So that by this intermeddling a portion of the placenta becomes detached, and profuse hæmorrhage follows, thus converting a natural into a preternatural case, and making manual interference necessary. Again, by delivering the secundines before the uterus has recovered from its fatigue, a secondary hæmorrhage may be induced. The organ may contract upon the introduction of the hand, and soon after the stimulus is removed may gradually expand, accompanied by a concealed hæmorrhage, thereby making a second introduction necessary. This, then, is meddlesome midwifery, and the introduction of the hand into the uterus under such circumstances, improper, worse than useless, and sometimes hazardous. The operation is always a serious affair, much dreaded by the patient, as well as by the feeling practitioner. It is productive of extreme suffering and fright to the woman, besides accompanied by the dangers above enumerated and others which I have not yet mentioned. Among them are rupture and inversion of the uterus. These accidents to an experienced man would not be very likely to happen, but they have occurred to the inexperienced. I myself met with this latter accident in the second year of my practice, under the following circumstances, and I relate the case for the good of others who may be similarly situated:

I was called in haste to see a Dutch woman in labor with her third child. It had been born a half hour previous to my arrival, and the attendants were all busily engaged in dressing or exulting over their trophy, while the poor mother lay neglected on a pallet, with shoulders elevated and feet to the fire, flooding profusely. With coat off and sleeves upturned, I proceeded to examine her. She at that moment began to gasp and fall into syncope. I immediately introduced my left hand into the uterus, and found the same relaxed and low down in a large pelvis. The placenta was wholly detached, except a portion about three inches in diameter, which was firmly adhering to the fundus. I attempted to detach it, but unsuccessfully, the uterus remained expanded, and I felt as though I could not separate it.

I slowly withdrew my hand, the motion of which caused a sensation of tenesmus, the woman made an expulsive effort and down came placenta, uterus and all, complete inversion! Here was danger both palpable and visible. Collecting my wits as quick as thought, I placed my folded hand against the presenting mass, and steadily and firmly reversed it. The uterus immediately contracted, expelling my hand and the separated placenta. The patient complained of very severe pain in one hip for a half hour after, which was assuaged by morphia. She recovered without any farther trouble; but my readers can better imagine my feelings for that day than I can describe them.

I however learned two things practically from the case: First, never to withdraw my hand while the uterus remained uncontracted; and secondly, to return the inverted uterus, if possible, without the least delay, not waiting to separate the adherent placenta.

For good advice on this subject, see Rigby.

Another evil which I think would sometimes accompany manual delivery of the placenta, if universally adopted, would be a more frequent occurrence of puerperal convulsions. But of this I cannot speak from facts; reason and analogy point to such a result. The cases where manual interference is necessary, are sufficiently numerous without resorting to it, when uncalled for. But these cases I will not now refer to; they are ably and amply treated by Moreau, Baudeloque, Dewees and Rigby. I will at this time only refer to two practical points in the stage under consideration.

The first is of minor importance, but still worthy of notice. I refer to tying the funis, whether one or two ligatures should be applied. Dewees recommends one only, and gives as a reason that "the evacuation from the open extremity of the cord will yield two or three ounces of blood, which favors the contraction of the uterus and expulsion of the placenta." Rigby is of the same opinion, and quotes Dewees for authority. Moreau and Baudeloque say there is no harm in applying two, and advise it if there should be hæmorrhage from the cord, while Murphy recommends two ligatures without assigning a reason. I began practice with one ligature, but have long since resorted to the application of two, and for the purpose of effecting the very same result as that sought by Dewees, viz., the "contraction of the uterus and the expulsion of the placenta." I believe that a full and rather firm placenta is more readily cast off than a flaccid and yielding one, from the fact that the breaking up of the attachment between the two surfaces is more easily effected by displacement. Besides, the larger the placenta the greater the stimulus it exerts upon the contracting uterus, favoring its expulsion. The other point is one I feel a little reluctance in presenting. I may be wrong in my views. I will venture to state them and let them go for what they will fetch, whether that be ridicule or neglect. It is concerning concealed hæmorrhage after complete delivery. During several months of the last year there was observed by physicians in this region of country, an unusual predisposition to hæmorrhage after delivery. I was often led to suspect that the supine position favored the occurrence of a concealed hæmorrhage instead of tending to prevent it. This at first view may appear a strange notion, but on reflection the idea may not seem so absurd. We know that there is always a tendency in the uterus, a short time after complete delivery, to expand. When examined through the parieties of the abdomen it no longer feels hard, but larger and softer. At the same time there is a slight flowing from the patulous vessels of the organ. Now if the head and shoulders are laid low, the pressure of the *viscera* is removed from the *fundus uteri*, a coagulum forms, distending the uterus longitudinally, hæmorrhage increases rapidly, and we soon find the fundus extending upward to the epigastrium, and the condition of the patient demanding an immediate manual delivery of the coagula before contraction, firm and efficient, can be induced. This has been my experience. Perhaps a semi-inclined position might be safer, until hæmorrhage actually occurs. I certainly would not recommend the erect position on any occasion, yet I have seen the placenta delivered from women in the standing posture by older hands than mine. In attempting to follow the example in a few cases, I soon found abundant reason to condemn the practice.

